# Autophagy-induced NR2F1 activation promotes the apoptosis of lens epithelial cells and facilitates cataract-associated fibrosis through targeting STAT3

**DOI:** 10.1016/j.gendis.2025.101549

**Published:** 2025-01-28

**Authors:** Hangjia Zuo, Xianyang Liu, Bingjing Lv, Ning Gao, Miaomiao Du, Xiang Gao, Yongguo Xiang, Rongxi Huang, Meiting Lin, Yakun Wang, Yonglin Chen, Hong Cheng, Tong Zhang, Shijie Zheng, Wenjuan Wan, Ke Hu

**Affiliations:** a The First Affiliated Hospital of Chongqing Medical University, Chongqing Key Laboratory of Prevention and Treatment on Major Blinding Diseases, Chongqing Eye Institute, Chongqing Branch (Municipality Division) of National Clinical Research Center for Ocular Diseases, Chongqing 400016, China; b Endocrinology, Chongqing General Hospital, Chongqing 400013, China; c Chongqing Medical University, Chongqing 400016, China

**Keywords:** Apoptosis, Autophagy, Cataracts, Epithelial-mesenchymal transition, JAK1/STAT3 pathway, NR2F1

## Abstract

Cataracts, a widely prevalent ocular pathology, engender visual impairment and emerge as a primary etiological factor contributing to ocular blindness. Substantial evidence substantiates that epithelial–mesenchymal transition stands prominently among the pivotal causative factors associated with this debilitating condition. However, the underlying mechanism remains unclear. In the present study, we analyzed the single-cell data and found that the mRNA expression of nuclear receptor subfamily 2 group F member 1 (NR2F1/COUP-TFI) was notably decreased in fibrocytes compared with epithelium. Interestingly, we observed a significant up-regulation of NR2F1 protein in the anterior subcapsular cataract mice model and transforming growth factor-β1 (TGF-β1)-treated SRA01/04 cells. Furthermore, we found that TGF-β1 stimulation disrupted the balance of autophagy, leading to impaired degradation and increased protein levels of NR2F1 in SRA01/04 cells. Subsequently, after anterior chamber injection of NR2F1 adeno-associated virus in anterior subcapsular cataract mice, the development of fibrosis was alleviated. *In vitro*, the knockdown of NR2F1 in SRA01/04 also mitigated the TGF-β1-induced epithelial–mesenchymal transition. Mechanically, NR2F1 proteins directly interacted with the promoter region of STAT3 and orchestrated the up-regulation of phosphorylated STAT3 (p-STAT3), thereby facilitating the apoptosis and migration of SRA01/04 cells via the JAK1/STAT3 pathway, resulting in epithelium fibrosis and cataracts. Furthermore, inhibition of p-STAT3 obviously attenuated apoptosis and fibrosis of SRA01/04 cells. Collectively, our study provides a novel therapeutic target for cataracts and offers insight into the underlying mechanism of the epithelial–mesenchymal transition of cataracts.

## Introduction

Cataracts, a prevalent ocular condition, result in visual impairment and primarily contribute to blindness.^1^^,^^2^ Over the past few years, there has been a significant increase in the prevalence of cataracts, which are characterized by impaired eyesight and a gradual decline in vision.^3^ The abnormal growth and movement of lens epithelial cells invading stromal cells are common conditions that contribute to the development of cataracts.^4^^,^^5^ In the process of epithelial–mesenchymal transformation (EMT), lens epithelial cells produce significant fibronectin 1 (FN1), α-smooth muscle actin (α-SMA), and vimentin (VIM) to obtain a mesenchymal cell phenotype. This is accompanied by an increase in the migratory ability of lens epithelial cells.^6^^,^^7^ While surgical interventions can effectively restore vision in individuals with cataracts, a significant number of patients experience persistent vision loss or pupil deformation within the ensuing 2–5 years after surgery. This phenomenon profoundly influences the quality of life of these patients.^8^ Hence, it is imperative to explore a different approach for treating cataracts.

Anterior subcapsular cataract (ASC) is an important type of cataract, characterized by a fibrotic plaque beneath the anterior lens capsule and is primarily induced by trauma or cytokine stimulation.^7^^,^^9^^,^^10^ Over the past few years, the demand for implantable lens surgery has steadily increased. Importantly, recent studies have recognized ASC as a complication that arises from this procedure.^11^ The connection between the origin of ASC and lens epithelial cells, particularly human lens epithelial cells, remaining after cataract surgery is widely recognized. The proliferation, migration, and mesenchymal transformation of these cells result in the posterior capsule appearing white and cloudy.^9^ The increase in transforming growth factor-β (TGF-β) inside ASC has been widely recognized as a crucial trigger, leading to EMT in lens epithelial cells. During this process, cells exhibit either migration towards the cell-free posterior capsule or the formation of fibrotic plaques.^12^^,^^13^ Consequently, inhibiting lens epithelial cell migration and proliferation through EMT modulation emerges as a promising therapeutic strategy for ASC treatment and prevention. Considerable advancements have been achieved in the development of medications and gene therapy that focus on the pathogenesis of ASC, utilizing epigenetic methods. Targeted preventive and therapeutic measures for these fibrotic cataracts have consequently become available.

Nuclear receptor subfamily 2 group F member 1 (NR2F1/COUP-TFI) acts as a transcriptional regulator for numerous genes and plays a crucial role in diverse biological processes such as cell growth, differentiation, and migration.^14^^,^^15^ Moreover, NR2F1 is implicated in several ophthalmic diseases; notably, it has been identified as the causative factor in Bosch–Boonstra–Schaaf optic atrophy syndrome, which often presents with optic atrophy, leading to vision impairment.^16^ New studies have provided insight into the important function of NR2F1 in the development of mesenchymal fibrosis in cancerous cells.^17^^,^^18^ A study conducted by Carolina et al revealed that during the early phases of breast cancer progression, the suppression of NR2F1 by human epidermal growth factor receptor 2 (HER2) aids in the spread of cancer cells by initiating EMT and activating a combination of luminal and basal-like characteristics.^19^ Nonetheless, the exact process through which NR2F1 functions in the transition from an epithelial state to a mesenchymal state in lens epithelial cells is still unknown.

The current investigation revealed that TGF-β1 stimulation disrupted autophagy, resulting in impaired degradation and elevated protein levels of NR2F1 in epithelial cells. In the *in vitro* environment, the inhibition of NR2F1 in lens epithelial cells reduced TGF-β1-induced proliferation, migration, and EMT. Additionally, NR2F1 adeno-associated virus (AAV)-infected ASC mice exhibited a deceleration in the progression of fibrosis. Mechanistically, dual-luciferase experiments revealed that NR2F1 bound directly to the promoter of signal transducer and activator of transcription 3 (STAT3) and regulated the expression of phosphorylated STAT3 (p-STAT3), promoting lens epithelial cell fibrosis, migration, and apoptosis and resulting in the development of cataracts. Collectively, these findings indicate that NR2F1 represents a promising therapeutic target for treating fibrotic cataracts.

## Methods and materials

### Cell culture and treatment

The SRA01/04 cell line was obtained from Procell Life Science & Technology Co., Ltd. and cultured in Dulbecco's modified Eagle medium (Gibco, Life Technologies, NY, USA) supplemented with 10% fetal bovine serum (Gibco, Life Technologies). The cells were maintained in an incubator at 37 °C with 5% CO_2_. For TGF-β1 treatment, cells were seeded in 6-well plates and treated with 10 ng/mL of TGF-β1 (PeproTech, Suzhou, China) for 48 h. The autophagy inhibitor chloroquine (T8689, TargetMol, USA) was applied at concentrations of 5, 10, 20, 40, and 80 μM. Additionally, the P-STAT3 inhibitor NSC 74859 (MCE, Shanghai, China) was added 24 h before TGF-β1 treatment. NR2F1 agonist 1 (HY-149913, MCE) was treated at 0.5 or 1 μM concentration.

### Lentiviral cell transduction

Lentiviral vectors were utilized to suppress NR2F1 expression in accordance with the manufacturer's recommended protocols. Briefly, 2 × 10^5^ cells per well were sowed in 6-well plates (Jet Biofil). Once the cells had adhered to the wall, they were infected with the lentiviral particles at a multiplicity of infection of 45 for 6 h. On the third day after transfection, the cells were examined and their fluorescence was visualized using a fluorescence microscope (DMIL4000, Leica, Germany) to confirm the efficiency of transduction. Subsequently, the stably transformed cell lines were selected through treatment with puromycin dihydrochloride at a concentration of 2 μg/mL.

### Adeno-associated virus injection

C57BL/6J mice were first induced into general anesthesia through intraperitoneal administration of ketamine. To facilitate ocular dilation, tropicamide was administered topically. The periocular region was then meticulously cleaned with povidone iodine to minimize the risk of infection. Under an operating microscope, the mice were carefully positioned. A Hamilton syringe, fitted with a 30-gauge needle, was prepared for precise injection. A minimally invasive incision was made in the cornea to gain access to the anterior chamber of the eye. Slow and controlled injections of either AAV-NC or AAV-NR2F1 were administered into the anterior chamber with caution to prevent damage to the corneal endothelium and iris. The needle was withdrawn slowly to minimize the backflow of the vector.^20^

### Real-time quantitative PCR

RNA extraction was performed using the TRIzol reagent (Roche, Swiss). After reverse transcription of the RNA into cDNA with the RT Master Mix (AG11705, Accurate Biotechnology (Hunan) Co., Ltd., Changsha, China), the cDNA was combined with the SYBR Green qPCR Master Mix in a light-protected environment (AG11708, Accurate Biotechnology (Hunan) Co., Ltd., Changsha, China). mRNA expression levels were assessed using the ABI 7500 Real-Time PCR System (Applied Biosystems, USA). The expression of β-actin was employed as an internal control to normalize the mRNA levels, which were quantified using the 2^−ΔΔCT^ method. Primers were synthesized by Shanghai Sangon Co., Ltd. The forward primer for NR2F1 was 5′-ATCGTGCTGTTCACGTCGTCAGAC-3′ and the reverse primer was 5′-TGGCTCCTCACGTACTCCTC-3′. For β-actin, the forward primer was 5′-GTGACGTTGACATCCGTAAAGA-3′ and the reverse primer was 5′-GCCGGACTCATCGTACTC-3′.

### Western blot

SRA01/04 cells or lenses were first rinsed twice with phosphate-buffered saline (PBS). Following this, pre-cooled RIPA buffer (R0020, Solarbio) was added to the cells to effectively lyse and extract total proteins. The protein concentration in the lysates was then determined using the Bicinchoninic Acid Kit (Beyotime, Shanghai). Subsequently, the protein was separated by gel electrophoresis and transferred onto a polyvinylidene fluoride membrane. Next, the membrane was obstructed using Fast Blocking Western (Yeasen, Shanghai) and left to incubate with the primary antibody overnight at a temperature of 4 °C. Finally, the bands were visualized by ECL kit (KF8001, Affinity) and quantified with Image J.^21^ The primary antibodies employed in this investigation are listed in Table S1.

### Immunofluorescence staining

Sh-NC or Sh-NR2F1 SRA01/04 cells were stimulated with TGF-β1 and incubated for 48 h. Following this treatment, the cells were fixed using paraformaldehyde at room temperature for 30 min. This was followed by permeabilization with 0.5% Triton X-100 for 10 min and then blocking with 1% bovine serum albumin for 1 h to reduce non-specific antibody binding. After the blocking step, the cells were incubated at 4 °C overnight in a humidity chamber with primary antibodies. The following day, cells were incubated with secondary antibodies. The secondary antibodies used were Alexa Fluor 488-labeled goat anti-rabbit IgG (H + L) and Cy3-labeled donkey anti-goat IgG (H + L), both diluted at a ratio of 1:500 (Beyotime). After rinsing with PBS, nuclear staining was performed using 4′,6-diamidino-2-phenylindole (DAPI). Images were conducted using a laser scanning confocal microscope (Leica, Germany).

Lens paraffin sections were subjected to fluorescent immunolabeling using specific primary antibodies to target proteins of interest. These were followed by the application of appropriate secondary antibodies to amplify the signal. Subsequent analysis of these labeled sections was performed using a fluorescence microscope (Leica, Germany).

### Hematoxylin and eosin staining

The lens tissue was fixed using buffered formalin to preserve its structure and then embedded in paraffin. Using a microtome, the paraffin-embedded lens blocks were sectioned into 5-micron-thick slices. These thin sections were carefully mounted onto glass slides. The slides underwent a deparaffinization process using xylene to remove the paraffin, followed by a rehydration sequence with graded alcohols ranging from 70% to 100%. The rehydrated sections were then stained using the conventional hematoxylin and eosin staining protocol which provides contrast by staining cell nuclei blue (hematoxylin) and cytoplasmic structures pink (eosin). Finally, the stained sections were examined and images were captured using a high-quality microscope (Leica, Germany).

### Masson staining

Lens sections were immersed in clearer for 10 min. This step was repeated two times, gently shaking off excess liquid between each step. The tissue sections were soaked in increasingly less concentrated ethanol solutions and finally soaked in distilled water to rehydrate the tissue. The tissue was soaked in absolute ethanol for 5 min, followed by 95% ethanol for 5 min, 85% ethanol for 5 min, and 75% ethanol for 5 min. The tissue was then rinsed with distilled water for 1 min. The dehydrated tissue sections were immersed in Bouin's solution or Zenker's solution overnight and then rinsed with running water. The sections were treated with Harris hematoxylin solution or iron hematoxylin for 5–10 min, followed by a gentle rinse with running water. Differentiation of the sections was carried out using 0.8%–1% hydrochloric acid alcohol, followed by washing with running water for several minutes. An alternative treatment involved the use of a lithium carbonate solution for a bluer hue, followed by washing with running water. Afterward, the sections were treated with a solution of ponceau acid fuchsin for 5–10 min, followed by rinsing with flowing water. Additional therapy involved being exposed to a solution of phosphomolybdic acid for approximately 5 min and then stained with a solution of aniline blue for 5 min without any rinsing. The sections were subsequently subjected to a 1-min treatment using 1% acetic acid and were then dehydrated multiple times using 95% alcohol. The tissue sections were dehydrated with absolute alcohol and transparentized with xylene, then mounted with neutral balsam, and examined under a microscope (Leica, Germany).

### ASC model

In this experiment, a group of 8-week-old C57BL/6J mice was divided into two equal groups. After dilating the pupils with the compound tolbutamide, the anterior capsule was scratched by puncturing the mice's cornea using a hollow needle with a diameter of 0.3 mm under a microscope. The depth of the needle was approximately 1 mm. Cataracts in the experimental group were the most obvious on the 7th day.

### Migration assay

Sh-NC or Sh-NR2F1 SRA01/04 cells were seeded and cultured in the upper chambers of 24-well Transwell plates (Corning, Inc.), with a seeding density of 5 × 10^4^ cells per well. After adhering to the chamber walls, the cells were treated with TGF-β1 for 48 h. The cells that had traversed the filter were then fixed using 4% paraformaldehyde for 10 min to ensure their immobilization, and stained with 1% crystal violet. Non-migrated cells on the upper side of the microporous membrane were removed using a cotton swab. The migrated cells on the lower side were visualized under a fluorescence microscope (Leica, Germany). The quantification of the migrated cells was accomplished using ImageJ software.

### Terminal deoxynucleotidyl transferase-mediated dUTP nick end labeling (TUNEL) staining

SRA01/04 cells were seeded into a 48-well plate with either Sh-NC or Sh-NR2F1 and subsequently exposed to TGF-β1 for 48 h. After that, the cells were rinsed with PBS, followed by fixation using 4% paraformaldehyde for 30 min. They were then washed again with PBS and treated with 0.3% Triton X-100 PBS at room temperature for 5 min. Afterward, the specimens were rinsed two times with PBS, and then 50 μL of TUNEL detection solution was introduced to the specimens, followed by incubation at 37 °C for 60 min in the absence of light. After applying an anti-fluorescence quenching sealing solution, the samples were examined using a fluorescence microscope.

Lens sections were initially treated with Protease K at 37 °C for 20 min to facilitate tissue permeabilization. Following the enzymatic treatment, the sections were thoroughly washed three times with PBS. Subsequently, the appropriate amount of terminal deoxynucleotidyl transferase (TdT) enzyme was added to the sections, and they were incubated at 37 °C for 1 h. To visualize the cell nuclei, the sections were stained with DAPI. The stained lens sections were examined and images were captured using a fluorescence microscope (Leica, Germany).

### Dual-luciferase assay

HEK293T cells were transfected with Luciferase reporter and Renilla luciferase vectors obtained from Wuhan GeneCreate Biological Engineering Co., Ltd., China. These cells were previously transfected with NR2F1 overexpressing plasmid and either STAT3 mutant or wild-type plasmid. This transfection was performed 24 h after seeding the cells into a 24-well plate (3 × 10^4^ cells per well) using Lipofectamine TM 2000 (Invitrogen, USA). The measurement of luciferase function was performed 24 h after transfection using a Dual-Luciferase Reporter Assay kit from Yeasen in Shanghai, China.

### Statistical analysis

The data was analyzed using a two-tailed *t*-test and one-way ANOVA. The results illustrated in the figures are indicative of three or more independent repetitions. Statistical significance was considered at a significance level of *p* < 0.05, and *p* values < 0.01 were regarded as highly significant.

## Results

### The protein level of NR2F1 is increased in the ASC model

**Figure 1:**
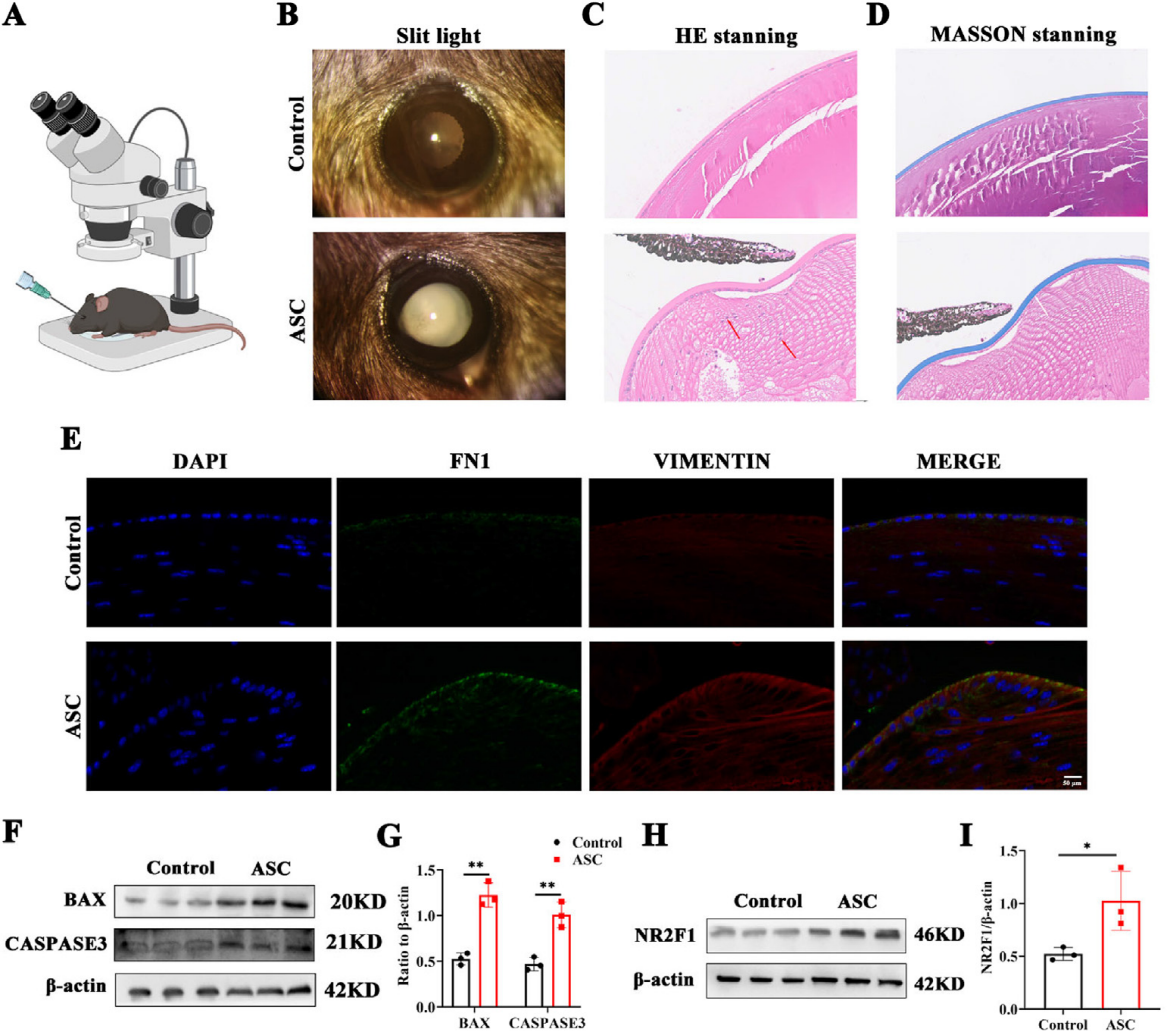
The protein level of NR2F1 is increased in the ASC model. **(A)** The anesthetized and dilated mice were positioned beneath a microscope for the ASC modeling procedure. **(B)** Cataract lesions in control and ASC mice using a slit lamp. **(C)** Hematoxylin and eosin staining in lens section of control and ASC mice. Scale bar, 100 μm. **(D)** Masson staining of the two groups. Scale bar, 100 μm. **(E)** The immunofluorescence results of FN1 and VIM in lens slides of control and ASC groups. Scale bar, 50 μm. **(F, G)** The protein expression and quantification of apoptosis-related markers BAX and CASP3 in control and ASC groups. *n* = 3 per group; mean ± standard deviation; ∗∗*p* < 0.01; unpaired student's *t*-test. **(H, I)** The protein level and quantitative chart of NR2F1 in the two groups mentioned above. *n* = 3 per group; mean ± standard deviation; ∗*p* < 0.05; unpaired student's *t*-test. NR2F1, nuclear receptor subfamily 2 group F member 1; ASC, anterior subcapsular cataract; FN1, fibronectin 1; VIM, vimentin; BAX, Bcl-2-associated X; CASP3, caspase 3.

To examine the involvement of NR2F1 in fibrotic cataracts, C57BL/6J mice were subjected to ASC induction (Fig. 1A). The use of this mouse model for lens anterior capsular injury is a widely accepted approach to investigate various aspects of lens epithelial cells, including cell death, cell movement, EMT, the accumulation of extracellular matrix components, and the development of subcapsular plaques.^22^, ^23^, ^24^ These features closely resemble the pathological characteristics observed in human ASC and posterior capsule opacification, with the peak of pathological manifestations occurring on the 7th day after modeling.^9^^,^^25^ Seven days after injury, slit lamp examination revealed opaque and cloudy lenses in the ASC group (Fig. 1B). Hematoxylin and eosin staining revealed a substantial proliferation of lens epithelial cells, which migrated towards the inner regions of the lens, adopting a radial pattern (Fig. 1C). Furthermore, Masson staining corroborated these findings, revealing a significant presence of collagen fibrils beneath the anterior capsule in ASC mice, indicative of fibrotic changes (Fig. 1D). Taken together, these findings suggest the successful development of an ASC mouse model, which exhibited an EMT process.

To further validate the fibrotic pathology inherent in the ASC model, we conducted an immunofluorescence analysis. The results showed a significant increase in the levels of fibrotic markers, such as FN1 and VIM, in the ASC group compared with the control group (Fig. 1E). Consistent with prior research highlighting the strong association between apoptosis and cataract disease, our findings also revealed a significant increase in ASC mice in the protein levels of apoptotic markers Bcl-2-associated X (BAX) and caspase 3 (CASP3) (Fig. 1F, G). Notably, the protein level of NR2F1 was substantially increased in the ASC group (Fig. 1H, I). Collectively, these observations imply a potential contributory role of NR2F1 in the development of ASC.

### TGF-β1 mediates the fibrosis process in human lens epithelial cells

**Figure 2:**
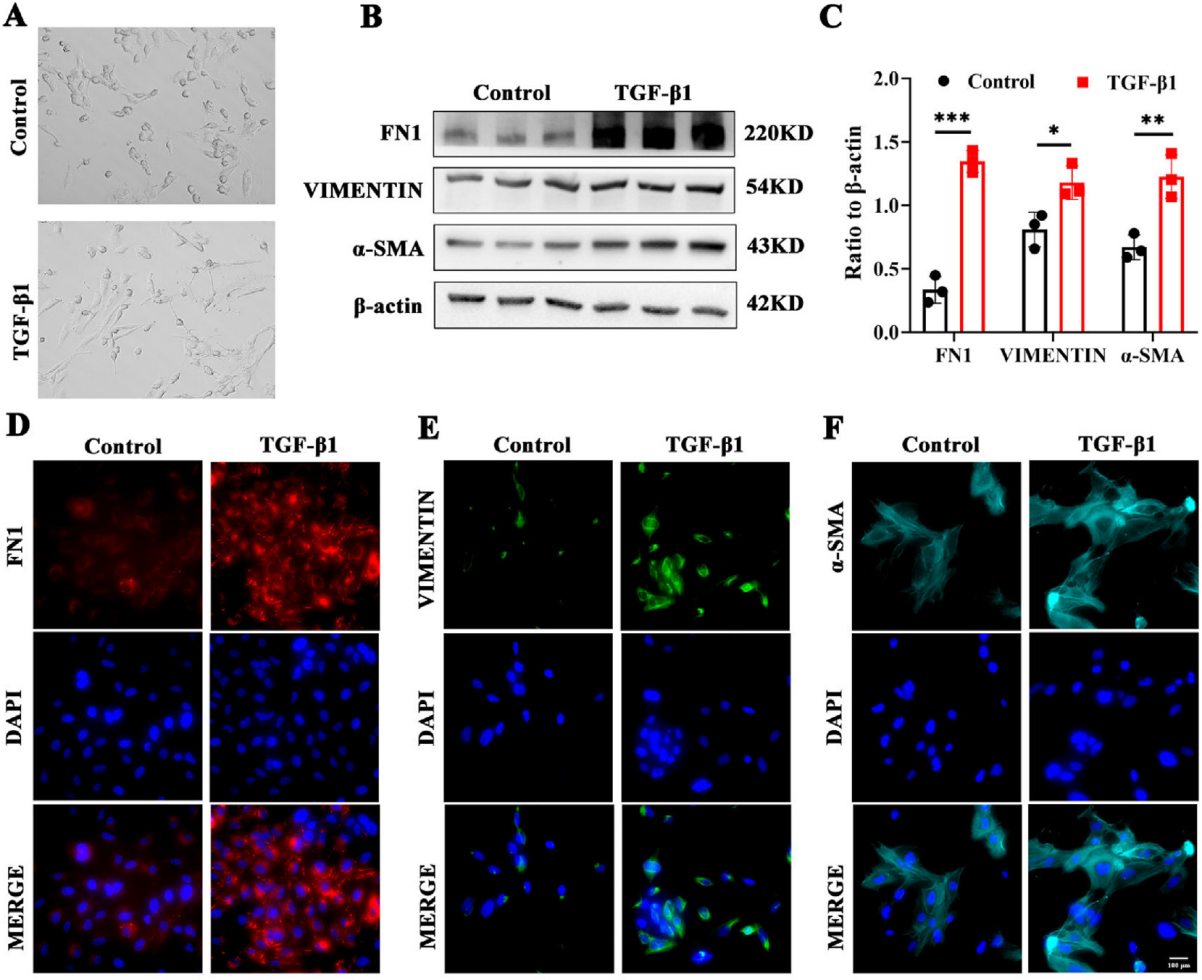
TGF-β1 mediates fibrosis of SRA01/04 cells. **(A)** Light field images of SRA01/04 cells treated with PBS or TGF-β1. **(B, C)** The protein expression and quantification of fibrosis-related markers FN1, VIM, and α-SMA in SRA01/04 cells with or without TGF-β1. *n* = 3 per group; mean ± standard deviation; ∗*p* < 0.05, ∗∗*p* < 0.01, ∗∗∗*p* < 0.001; unpaired student's *t*-test. **(D**–**F)** Immunofluorescence of FN1, VIM, and α-SMA in the two groups mentioned above. Scale bar, 100 μm. TGF-β1, transforming growth factor-β1; PBS, phosphate-buffered saline; FN1, fibronectin 1; α-SMA, α-smooth muscle actin; VIM, vimentin.

Due to the small size and difficulty in isolating primary lens cells, we decided to employ the widely used lens cell line SRA01/04 for further experiments. Following 48 h of exposure to TGF-β1, notable morphological changes were observed in the cells, characterized by an increase in size, a sparse cytoplasm, and an elongated pike shape (Fig. 2A). Following Western blot analysis, an evident increase in the protein levels of the mesenchymal markers FN1, VIM, and α-SMA was observed in the TGF-β1-induced SRA01/04 cells (Fig. 2B, C). Immunofluorescence analysis also revealed increased levels of FN1, VIM, and α-SMA in human lens epithelial cells subjected to TGF-β1 treatment compared with those in the control group (Fig. 2D–F).

### TGF-β1 mediated autophagy dysfunction results in increased protein levels of NR2F1

**Figure 3:**
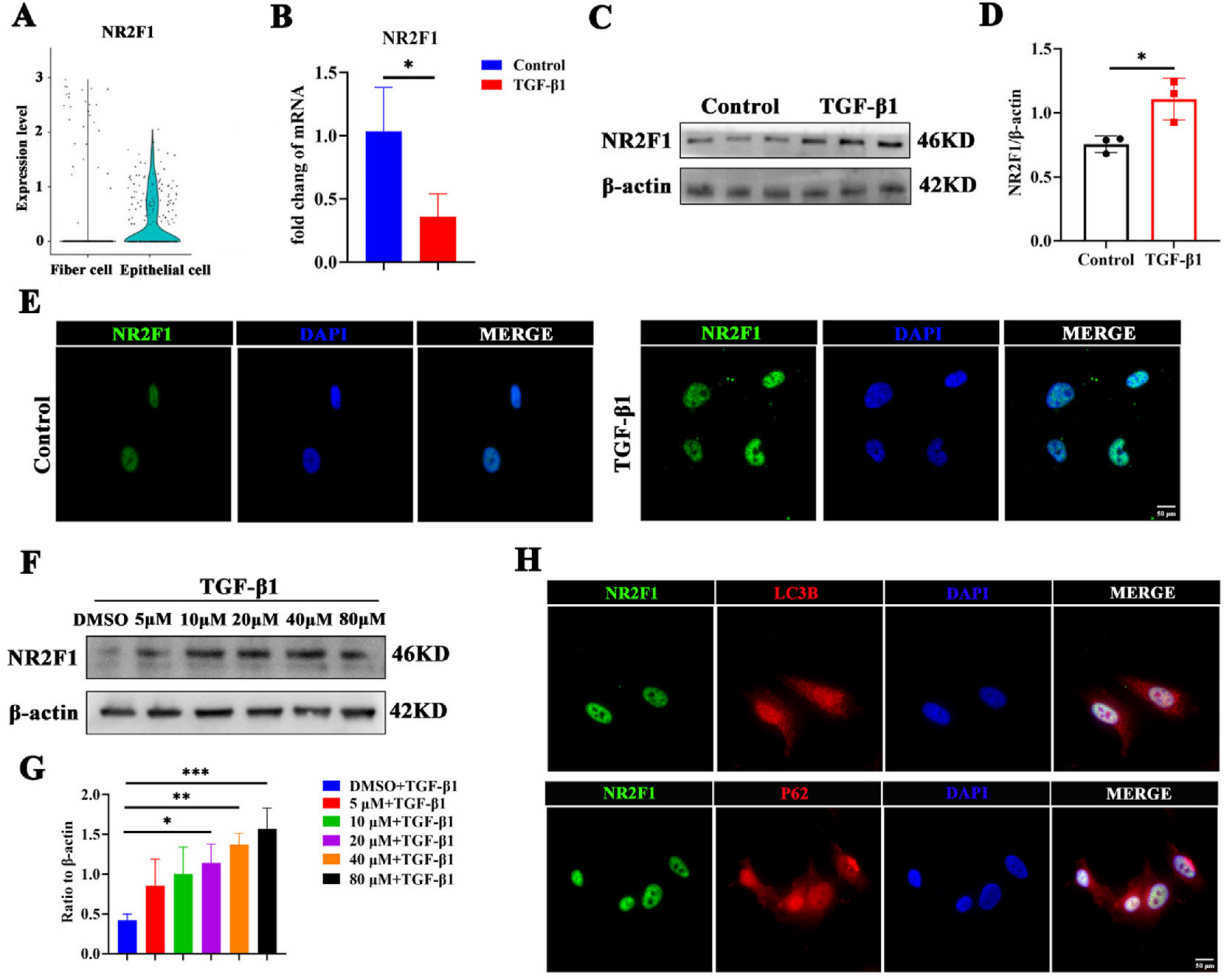
TGF-β1 mediated autophagy dysfunction resulting in an increased protein level of NR2F1. **(A)** The mRNA profile of NR2F1 in lens epithelial cells and fiber cells using single-cell data. **(B)** The mRNA level of NR2F1 in SRA01/04 cells with PBS or TGF-β1. *n* = 3 per group; mean ± standard deviation; ∗*p* < 0.05; unpaired student's *t*-test. **(C, D)** The protein expression and quantification of NR2F1 in TGF-β1-induced SRA01/04 cells. *n* = 3 per group; mean ± standard deviation; ∗*p* < 0.05; unpaired student's *t*-test. **(E)** Subcellular location of NR2F1 in PBS- or TGF-β1-treated SRA01/04 cells. Scale bar, 50 μm. **(F, G)** The protein level and quantification of NR2F1 in SRA01/04 cells stimulated with the autophagy inhibitor chloroquine at concentrations of 5, 10, 20, 40, and 80 μM, respectively. *n* = 3 per group; mean ± standard deviation; ∗*p* < 0.05, ∗∗*p* < 0.01, ∗∗∗*p* < 0.001; one-way ANOVA. **(H)** Co-localization of LC3B and NR2F1 along with P62 and NR2F1 in SRA01/04 cells with TGF-β1. Scale bar, 50 μm. TGF-β1, transforming growth factor-β1; NR2F1, nuclear receptor subfamily 2 group F member 1; PBS, phosphate-buffered saline; LC3B, microtubule-associated protein 1 light-chain 3B.

To investigate the influence of NR2F1 on the development of fibrosis, we analyzed single-cell information obtained from the embryonic eye dataset (GSE228370). Compared with those in epithelial cells, NR2F1 mRNA levels in fiber cells were lower (Fig. 3A). These findings are consistent with the results obtained in TGF-β1-treated SRA01/04 cells (Fig. 3B). Interestingly, the protein expression of NR2F1 was notably increased in SRA01/04 cells following treatment with TGF-β1 (Fig. 3C, D). Immunofluorescence analysis also revealed an increase in the protein level of NR2F1 in the TGF-β1-treated SRA01/04 cells (Fig. 3E). This inconsistency in mRNA and protein expression deserves further exploration.

Previous studies have suggested that increased mRNA levels with decreased protein levels may be attributed to unbalanced protein degradation.^26^^,^^27^ The importance of autophagy in the development of fibrosis facilitates the essential stages of protein breakdown.^28^, ^29^, ^30^ Therefore, we next stimulated SRA01/04 cells with the autophagy inhibitor chloroquine at concentrations of 5, 10, 20, 40, and 80 μM. Western blot analysis revealed that NR2F1 protein level was increased after autophagy was inhibited (Fig. 3F, G). Immunofluorescence analysis also revealed an increase in the protein level of NR2F1 after autophagy was inhibited (Fig. S1). Furthermore, immunofluorescence analysis revealed colocalization of microtubule-associated protein 1 light-chain 3B (LC3B) and NR2F1 along with P62 and NR2F1 (Fig. 3H). These findings suggest that the suppression of autophagy in epithelial cells caused by TGF-β1 may be responsible for the increased protein level of NR2F1.

### Knockdown of NR2F1 significantly attenuates fibrosis both *in vivo* and *in vitro*

**Figure 4:**
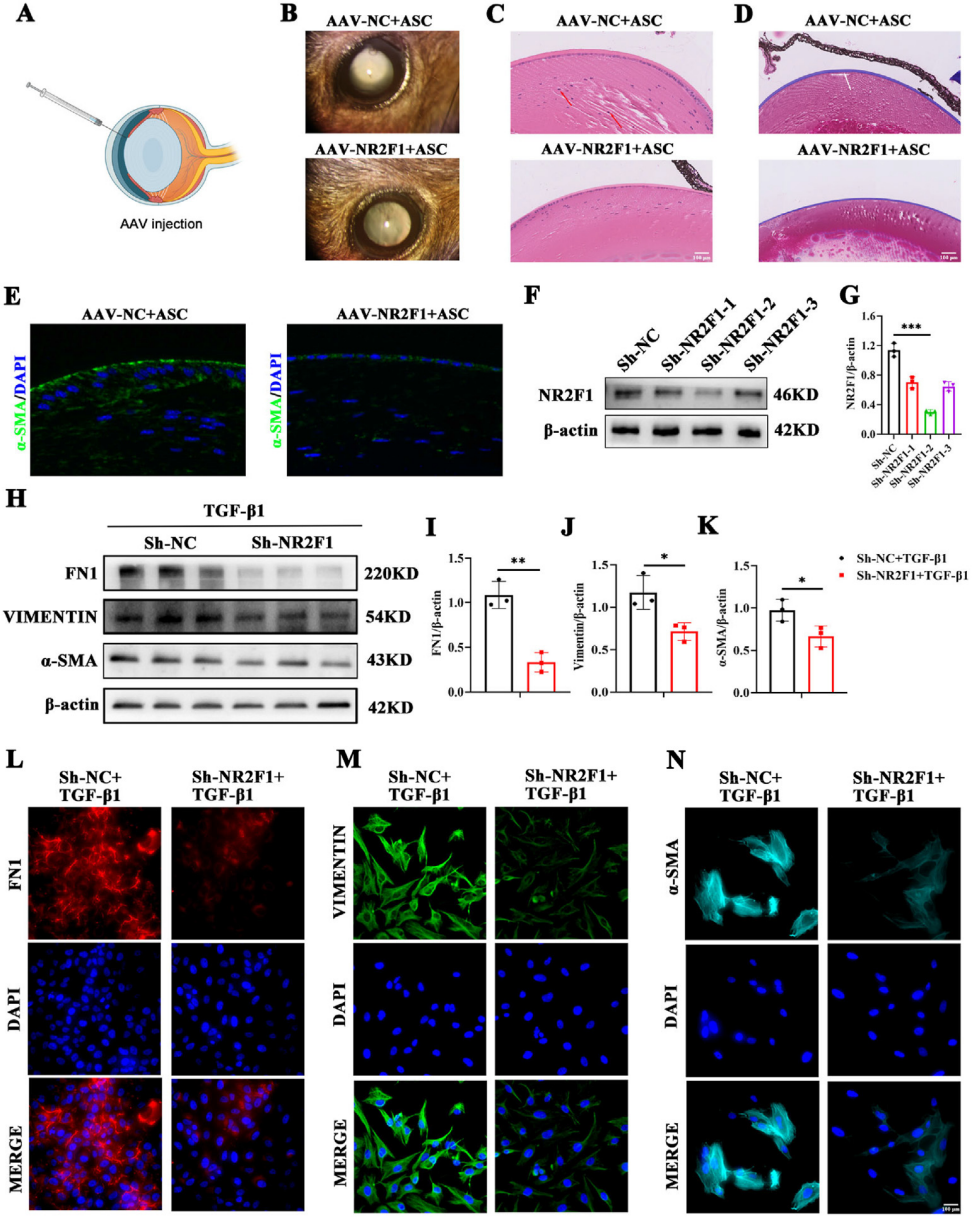
Knockdown of NR2F1 significantly attenuates fibrosis both *in vivo* and *in vitro*. **(A)** Adeno-associated adenovirus (AAV) was administered into the anterior chamber of mouse eyes. **(B)** Representative pictures of the ASC modeling group versus the negative control group under a slit lamp following AAV injection. **(C)** Hematoxylin and eosin staining in the two groups mentioned above. Scale bar, 100 μm. **(D)** Masson staining in the two groups. Scale bar, 100 μm. **(E)** The immunofluorescence intensity of α-SMA in the lens sections of AAV-NC and AAV-NR2F1 ASC mice. Scale bar, 50 μm. **(F, G)** The protein level and quantification of NR2F1 in SRA01/04 cells transfected with Sh-NC, Sh-NR2F1-1, Sh-NR2F1-2, or Sh-NR2F1-3 lentivirus. n = 3 per group; mean ± standard deviation; ∗∗∗*p* < 0.001; one-way ANOVA. **(H–K)** The protein expression and quantitative graphs of FN1, VIM, and α-SMA in TGF-β1-induced SRA01/04 cells with Sh-NC or Sh-NR2F1. *n* = 3 per group; mean ± standard deviation; ∗*p* < 0.05, ∗∗*p* < 0.01; unpaired student's *t*-test. **(L**–**N)** Immunofluorescence images of FN1, VIM, and α-SMA in TGF-β1-mediated SRA01/04 cells with Sh-NC or Sh-NR2F1. Scale bar, 100 μm. NR2F1, nuclear receptor subfamily 2 group F member 1; ASC, anterior subcapsular cataract; FN1, fibronectin 1; α-SMA, α-smooth muscle actin; VIM, vimentin; TGF-β1, transforming growth factor-β1.

To further explore the role of NR2F1 in the fibrosis process, AAVs carrying NR2F1 constructs were synthesized and administered via injection into the anterior chamber of the lens (Fig. 4A). Two weeks after AAV infection, the efficiency of NR2F1 knockdown was assessed, revealing a significant reduction in the protein levels of NR2F1 in the lenses of mice treated with NR2F1 AAVs (Fig. S2). Subsequently, mice were treated with AAV-NC (negative control) or AAV-NR2F1 and then subjected to the ASC modeling. Slit lamp examination revealed that mice treated with AAV-NR2F1 exhibited a milder degree of cataracts compared with their AAV–NC–treated counterparts (Fig. 4B). Hematoxylin and eosin staining revealed that the proliferation and migration abilities of lens epithelial cells in the AAV-NR2F1 group were reduced compared with those in the AAV-NC group (Fig. 4C). Masson staining revealed that the proliferative status of collagen fibers beneath the anterior capsule was less severe in AAV-NR2F1+ASC mice than in control mice (Fig. 4D). To further validate the observed phenotype, we conducted α-SMA immunofluorescence analysis on the aforementioned groups. The results revealed a decrease in fluorescence intensity in AAV-NR2F1+ASC mice, indicating a reduction in the extent of fibrosis. Collectively, these data indicate that inhibiting NR2F1 *in vivo* significantly attenuated the progression of fibrosis (Fig. 4E).

Additionally, NR2F1 lentiviruses were synthesized and introduced into SRA01/04 cells. Western blot analysis revealed that among the various constructs, Sh-NR2F1-2 exhibited the most pronounced silencing efficacy (Fig. 4F, G). Consequently, Sh-NR2F1-2 was selected for use in subsequent experiments. After the knockdown of NR2F1 in SRA01/04 cells treated with TGF-β1, Western blot analysis was conducted, revealing a significant decrease in the protein levels of the EMT markers FN1, VIM, and α-SMA (Fig. 4H–K). Moreover, immunofluorescence staining also showed decreased levels of FN1, VIM, and α-SMA (Fig. 4L–N). These findings indicated that the extent of fibrosis was significantly diminished both *in vitro* and *in vivo* following the knockdown of NR2F1.

### Knockdown of NR2F1 suppresses epithelial cell apoptosis and migration

**Figure 5:**
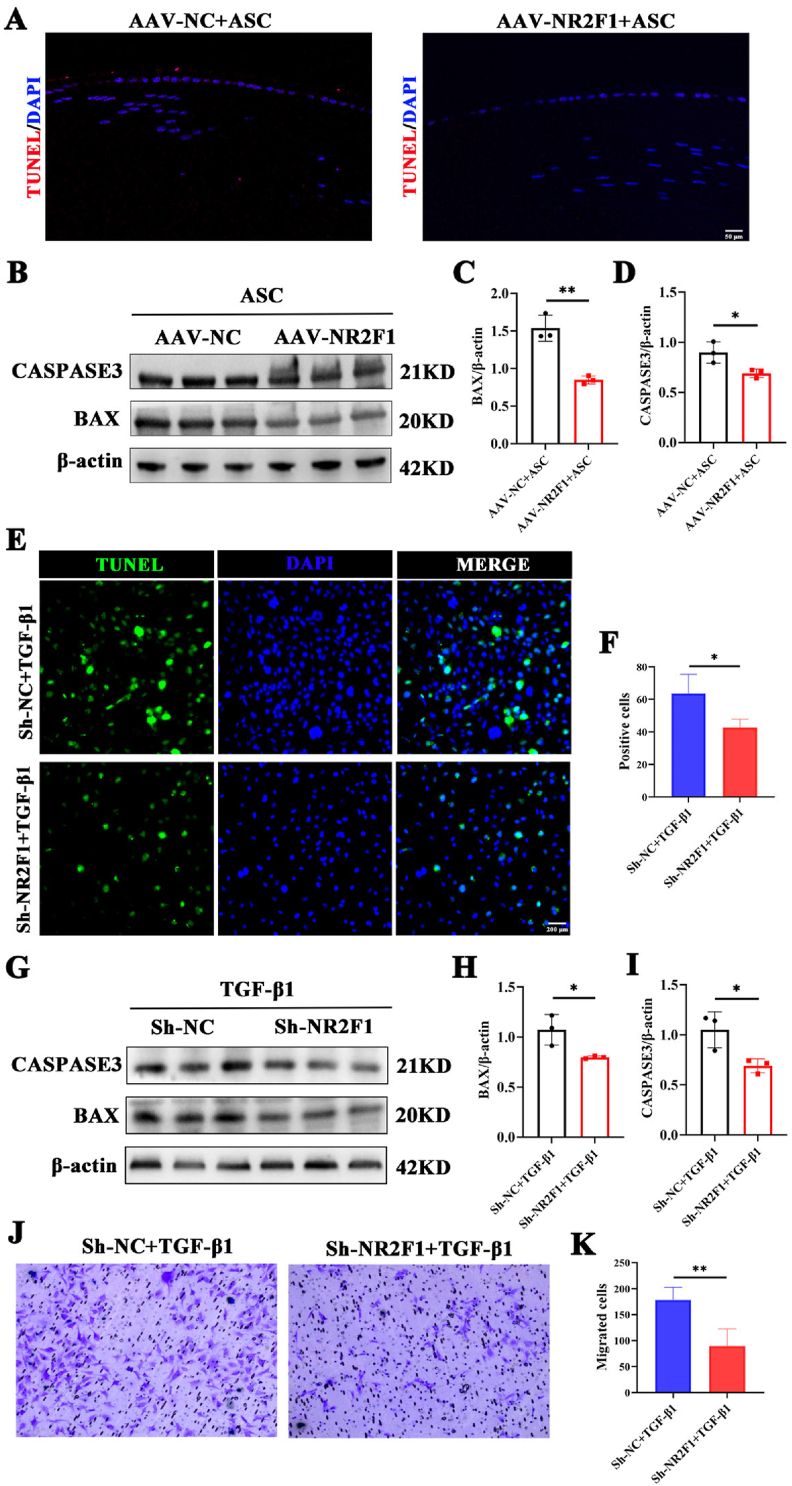
NR2F1 inhibition suppresses epithelial cell apoptosis and migration. **(A)** The TUNEL staining in lens sections of AAV-NC and AAV-NR2F1 mice with ASC. Scale bar: 50 μm. **(B**–**D)** The protein levels and quantitative charts of BAX and CASP3 in the two groups mentioned above. *n* = 3 per group; mean ± standard deviation; ∗*p* < 0.05, ∗∗*p* < 0.01; unpaired student's *t*-test. **(E, F)** The TUNEL staining in the TGF-β1-mediated group with Sh-NR2F1 compared with that with Sh-NC. *n* = 3 per group; mean ± standard deviation; ∗*p* < 0.05; unpaired student's *t*-test; scale bar, 200 μm. **(G**–**I)** The protein expression and quantification of BAX and CASP3 in TGF-β1-induced SRA01/04 cells with or without Sh-NR2F1. *n* = 3 per group; mean ± standard deviation; ∗*p* < 0.05; unpaired student's *t*-test. **(J, K)** The transwell assay in the two groups mentioned above. *n* = 4 per group; mean ± standard deviation; ∗∗*p* < 0.01; unpaired student's *t*-test. **(I–K)** The protein expression and quantification of BAX and CASP3 in TGF-β1-induced SRA01/04 cells with or without Sh-NR2F1. *n* = 3 per group; mean ± standard deviation; ∗*p* < 0.05; unpaired student's *t*-test. NR2F1, nuclear receptor subfamily 2 group F member 1; AAV, adeno-associated adenovirus; ASC, anterior subcapsular cataract; BAX, Bcl-2-associated X; CASP3, caspase 3; TGF-β1, transforming growth factor-β1.

Excessive apoptosis and migration of lens epithelial cells contribute to the development of cataracts.^30^, ^31^, ^32^ Therefore, the TUNEL assay revealed a decrease in positively stained cells within the lens sections of the AAV-NR2F1+ASC mice compared with those of the AAV-NC + ASC mice (Fig. 5A). Furthermore, Western blot analysis revealed significant reductions in the expression of the apoptosis markers CASP3 and BAX in ASC mice treated with AAV-NR2F1 (Fig. 5B–D). *In vitro* studies employing the TUNEL assay also revealed a reduction in apoptosis among Sh-NR2F1-treated SRA01/04 cells stimulated with TGF-β1 (Fig. 5E, F). Additionally, western blotting confirmed that the levels of the apoptosis markers CASP3 and BAX were significantly decreased in Sh-NR2F1-treated SRA01/04 cells exposed to TGF-β1 (Fig. 5G–I). The transwell assay results suggested that the migration capability was reduced in the Sh-NR2F1 group treated with TGF-β1 (Fig. 5J, K). In summary, the data collectively suggest that the knockdown of NR2F1 effectively inhibits apoptosis and migration in lens epithelial cells.

### NR2F1 directly binds to STAT3 and regulates its phosphorylation expression

**Figure 6:**
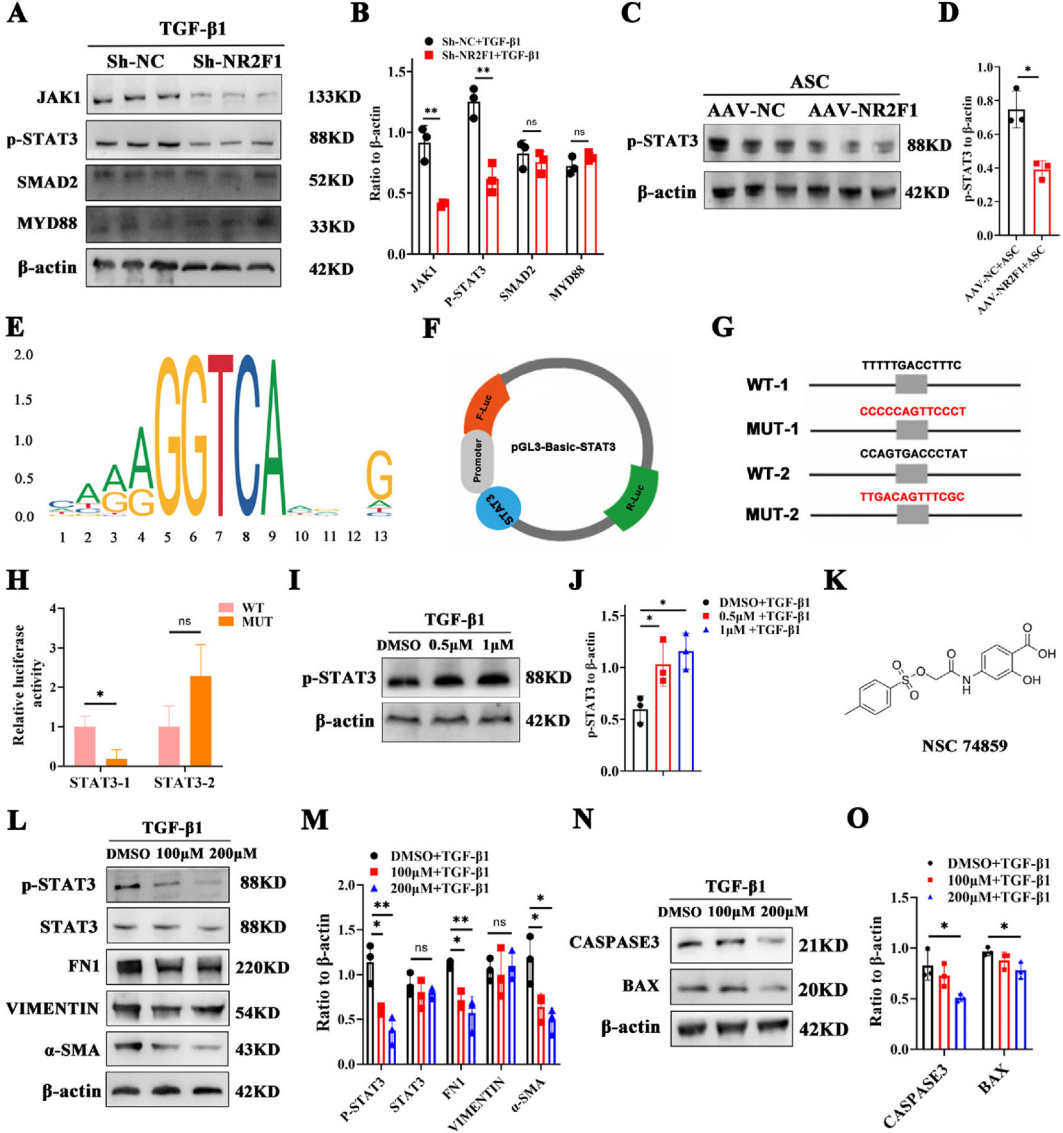
NR2F1 directly binds to STAT3 and regulates the expression of p-STAT3. **(A, B)** The protein levels and quantification of JAK1, p-STAT3, SMAD2, and MYD88 in TGF-β1-treated SRA01/04 cells with or without Sh-NR2F1. *n* = 3 per group; mean ± standard deviation; ns, >0.05; ∗∗*p* < 0.01; unpaired student's *t*-test. **(C, D)** The protein level and quantitative chart of p-STAT3 in AAV-NC or AAV-NR2F1 ASC mice. *n* = 3 per group; mean ± standard deviation; ∗*p* < 0.01; unpaired student's *t*-test. **(E)** The motif of NR2F1 predicted by the JASPAR website. **(F)** A dual-luciferase vector. **(G)** Mutant and wild-type STAT3 plasmids consequences. **(H)** The dual-luciferase assay for NR2F1 and STAT3 promoter. *n* = 3 per group; mean ± standard deviation; ns, >0.05; ∗*p* < 0.05; unpaired student's *t*-test. **(I, J)** The protein level of p-STAT3 in TGF-β1-induced SRA01/04 cells following NR2F1 agonist treatment at 0.5 or 1 μM. *n* = 3 per group; mean ± standard deviation; ns > 0.05; ∗*p* < 0.05; one-way ANOVA. **(K)** Structure of p-STAT3 specific inhibitor NSC 74859. **(L, M)** The protein expression and quantification of p-STAT3, STAT3, JAK1, FN1, VIM, and α-SMA in TGF-β1-induced SRA01/04 cells treated with the specific P-STAT3 inhibitor. *n* = 3 per group; mean ± standard deviation; ns, >0.05, ∗*p* < 0.05, ∗∗*p* < 0.01; one-way ANOVA. **(N, O)** The protein expression of the apoptosis-related markers BAX and CASP3 in TGF-β1-induced SRA01/04 cells treated with NSC 74859. *n* = 3 per group; mean ± standard deviation; ∗*p* < 0.05; one-way ANOVA. NR2F1, nuclear receptor subfamily 2 group F member 1; STAT3, signal transducer and activator of transcription 3; p-STAT3, phosphorylated STAT3; ASC, anterior subcapsular cataract; FN1, fibronectin 1; α-SMA, α-smooth muscle actin; VIM, vimentin; BAX, Bcl-2-associated X; CASP3, caspase 3; TGF-β1, transforming growth factor-β1; JAK1, Janus kinase 1; SMAD2, SMAD family member 2; AAV, adeno-associated adenovirus.

To elucidate the mechanisms underlying NR2F1 influencing epithelial cell fibrosis, we conducted a comprehensive review of the relevant literature. Notably, Janus kinase 1 (JAK1), SMAD family member 2 (SMAD2), MYD88, and p-STAT3 are strongly associated with the development of cataracts as well as some fibrotic diseases.^33^, ^34^, ^35^, ^36^ Subsequently, Western blot analysis was employed to screen for potential downstream pathways influenced by NR2F1. Compared with those in the control group, the protein levels of JAK1 and p-STAT3 in the Sh-NR2F1 group were significantly lower (Fig. 6A, B). Furthermore, *in vivo* research also suggested a decrease in the level of p-STAT3 in AAV-NR2F1-mediated ASC mice (Fig. 6C, D).

Considering the significant role of the STAT3 pathway in both fibrotic diseases and cancer metastasis during EMT,^37^, ^38^, ^39^, ^40^, ^41^, ^42^ we proceeded to explore the complex connection between NR2F1 and STAT3. Using the JASPAR website, we made predictions about potential target genes and discovered NR2F1 as a possible candidate that could directly attach to the STAT3 promoter, as shown in its displayed motif (Fig. 6E). We subsequently constructed a dual-luciferase vector (Fig. 6F) containing both mutant and wild-type STAT3 plasmids (Fig. 6G). In HEK293T cells co-transfected with NR2F1-overexpressing plasmids and STAT3-overexpressing plasmids, the dual-luciferase assay revealed that NR2F1 indeed directly bound to site 1 of the STAT3 promoter (Fig. 6H). Moreover, to evaluate the significance of STAT3 signaling, we treated SRA01/04 cells with an NR2F1 agonist. We subsequently conducted a Western blot assay to investigate the level of STAT3 phosphorylation, a critical marker for the activation of the STAT3 signaling pathway. The results showed that the phosphorylation of STAT3 was increased in SRA01/04 cells treated with TGF-β1 in conjunction with the NR2F1 agonist (Fig. 6I, J).

While STAT3 signaling has been thoroughly examined in cancer and fibrotic diseases,^43^^,^^44^ its involvement in lens epithelial cells and EMT remains predominantly unexplored. Subsequently, we explored the potential role of STAT3 signaling in TGF-β1-induced EMT in lens epithelial cells. The SRA01/04 cells were treated with NSC 74859, a specific inhibitor of p-STAT3. Following TGF-β1 stimulation, a Western blot analysis was conducted, which showed a marked reduction in the protein levels of p-STAT3, as well as significant decreases in the expression levels of FN1, VIM, and α-SMA (Fig. 6K–M). Furthermore, inhibition of p-STAT3 resulted in a reduction in the expression levels of BAX and CASP3 (Fig. 6N, O).

**Figure 7:**
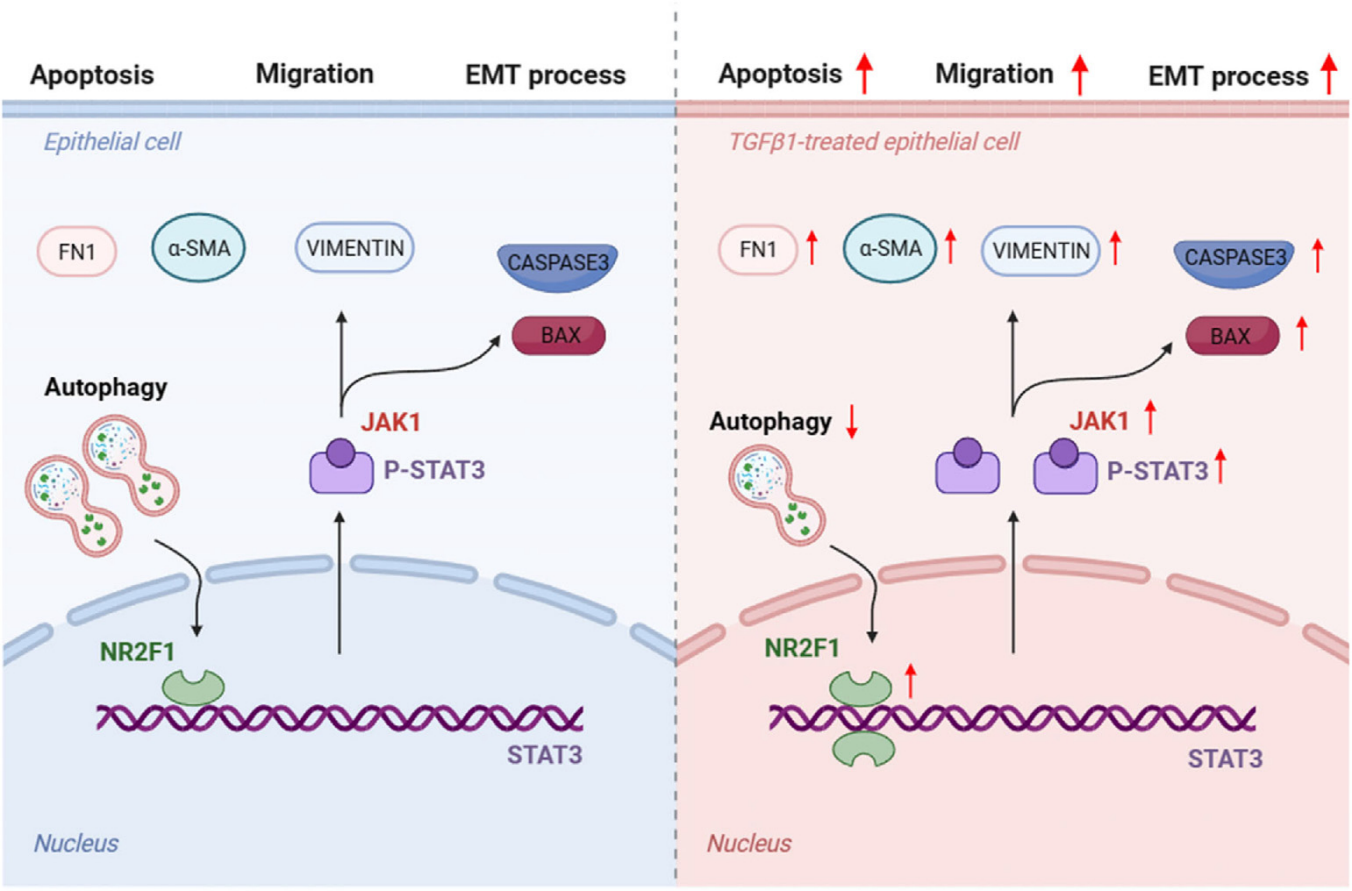
The regulatory mechanism of NR2F1 in TGF-β1-induced SRA01/04 cells. NR2F1, nuclear receptor subfamily 2 group F member 1; TGF-β1, transforming growth factor-β1.

In conclusion, our findings indicate that autophagy is impaired in epithelial cells upon TGF-β1 stimulation, which results in an up-regulation of the protein level of NR2F1. Furthermore, NR2F1 can directly interact with the promoter region of the STAT3 gene, thereby promoting the transcription of STAT3. This, in turn, promotes the progression of fibrosis and enhances lens epithelial cell migration and apoptosis, ultimately contributing to the development of cataracts (Fig. 7).

## Discussion

Fibrosis manifests in nearly all tissues and organs and often compromises organ functions, leading to significant morbidity and mortality. Aberrant EMT has been extensively documented as intricately associated with the fibrotic process, in which it instigates the cellular alterations essential for increased extracellular matrix production. This, in turn, substantiates the pivotal role of abnormal EMT in the pathogenesis of fibrosis across diverse tissues and organs.^45^, ^46^, ^47^ In this study, we analyzed a single-cell dataset of the embryonic eye and observed a notable reduction in the mRNA expression of NR2F1 in epithelial cells compared with that in fiber cells. In addition, the protein expression level of NR2F1 was significantly increased in both the ASC model and the *in vitro* TGF-β1-induced model, suggesting its potential association with fibrotic cataracts.

NR2F1 is a nuclear hormone receptor and transcriptional regulator, and its encoded protein acts as a homodimer and binds to 5′-AGGTCA-3′ repeats. Mutations in this gene have been implicated as causative factors in Bosch-Boonstra optic atrophy syndrome.^48^^,^^49^ Qiu et al reported that NR2F1 was predominantly associated with immunosuppressive cancer-associated fibroblast infiltration, and *in vitro* experiments revealed that NR2F1 knockdown could suppress cell migration and invasion through the EMT pathway in ovarian cancer patients.^50^ In the present study, silencing NR2F1 in human lens epithelial cells not only alleviated mesenchymal and apoptosis-related characteristics but also restrained EMT induced by TGF-β1. Furthermore, the severity of cataracts was diminished upon treatment with AAV-NR2F1 in the lens anterior capsules of injury-induced ASC mice. This observation underscores the therapeutic potential of NR2F1 in the context of fibrotic cataracts.

As a nexus for multiple oncogenic signaling pathways, STAT3 assumes a pivotal role in orchestrating the anti-tumor immune response.^51^ STAT3 functions as a prominent upstream mediator in the orchestration of EMT, demonstrating the capacity to instigate EMT-mediated metastasis in diverse malignancies, including brain tumors, thoracic cancers (encompassing lung cancer), and gastrointestinal cancers.^52^, ^53^, ^54^, ^55^, ^56^ Shen et al confirmed that blocking ATM inhibited EMT and reduced metastasis in cisplatin-resistant lung cancer cells via the JAK/STAT3/PD-L1 pathway.^57^ Moreover, Wang et al reported that SH2B3 impeded the acquisition of anoikis resistance and the advancement of EMT in lung cancer cells by suppressing the JAK2/STAT3 and SHP2/Grb2/PI3K/AKT signaling cascades.^54^ To further explore the downstream mechanisms of NR2F1, we screened pathways and observed a significant reduction in p-STAT3. Our findings, which stem from both *in vitro* cell cultures and an injury-induced ASC *in vivo* model, underscore the indispensable role of the STAT3 pathway in the EMT of human lens epithelial cells and fibrotic cataracts. Furthermore, NR2F1 could directly interact with the promoter region of STAT3, significantly enhancing lens epithelial cell migration, proliferation, and the progression of EMT. As a result, this mechanism contributes to the development of injury-induced ASC in mice. Notably, we revealed a previously unrecognized mechanism underlying NR2F1 promoting EMT through the positive regulation of the p-STAT3 signaling pathway.

There are also some limitations in this study. Although TGF-β1 is widely recognized as a key mediator in the induction of EMT, a process that is central to fibrosis in cataracts,^58^ some differences exist between *in vitro* models and *in vivo* cataract models, particularly in terms of the complexity and microenvironmental interactions. In future studies, we intend to isolate primary lens epithelial cells for more in-depth investigation. Additionally, we explored the effect of NR2F1 in an animal model and cells; however, the role of NR2F1 in cataract patients remains unclear. More studies should be performed to clarify its exact function in clinical patients. It is essential to acknowledge the intricate and multifaceted nature of cataracts, which encompass diverse subtypes with complex etiologies. However, our investigation focused primarily on the role of NR2F1 in a specific subset of cataracts, and other prevalent types remain unexplored. Future studies in this line of inquiry should extend their scope to investigate the influence of the NR2F1 gene on various cataract subtypes.

In conclusion, our study suggests that NR2F1 is related to EMT in cataracts. After NR2F1 silencing, lens opacity was reduced in the ASC model. Additionally, NR2F1 inhibited SRA01/04 cell migration, apoptosis, and EMT. Mechanistically, dual-luciferase experiments revealed that NR2F1 bound directly to the promoter of STAT3 and regulated the expression of p-STAT3, resulting in the development of cataracts. This discovery contributes valuable insights into potential therapeutic strategies for addressing pathological processes in the lens, with implications for conditions characterized by fibrosis and apoptosis.

## Ethics declaration

The approval for all experiments involving animals was obtained from the Ethics Committee of the First Affiliated Hospital of Chongqing Medical University.

## Funding

This work was supported in part by grants from the 10.13039/501100001809National Natural Science Foundation of China (No. 81870650, 82371098, 81970832, 81900885), the Natural Science Foundation Project of Chongqing, China (No. cstc2021jcyj-msxm3178), the Project Foundation of Chongqing Science and Technology Commission of China (No. cstc2021jscx-gksb-N0017, cstc2020jcyj-msxmX0829, cstc2021jcyj-msxmX0967, CSTB2022NSCQ-MSX1561)and the 10.13039/501100012166National Key Research and Development Program of China (No. 2020YFC2008200, 2020YFC2008204).

## CRediT authorship contribution statement

**Hangjia Zuo:** Conceptualization, Data curation, Formal analysis, Investigation, Methodology, Software, Validation, Writing – original draft. **Xianyang Liu:** Conceptualization, Data curation, Formal analysis, Methodology, Software, Validation, Writing – original draft. **Bingjing Lv:** Data curation, Supervision. **Ning Gao:** Data curation, Methodology, Software. **Miaomiao Du:** Data curation, Formal analysis, Software. **Xiang Gao:** Data curation, Formal analysis, Software. **Yongguo Xiang:** Data curation, Investigation, Methodology, Visualization. **Rongxi Huang:** Data curation, Formal analysis, Visualization. **Meiting Lin:** Investigation, Methodology, Software. **Yakun Wang:** Data curation, Formal analysis, Software. **Yonglin Chen:** Data curation, Formal analysis, Methodology. **Hong Cheng:** Data curation. **Tong Zhang:** Methodology, Software. **Shijie Zheng:** Data curation. **Wenjuan Wan:** Conceptualization, Data curation, Funding acquisition, Resources, Supervision, Validation, Writing – original draft, Writing – review & editing. **Ke Hu:** Conceptualization, Data curation, Funding acquisition, Investigation, Methodology, Project administration, Resources, Supervision, Validation, Writing – original draft, Writing – review & editing.

## Data availability

All data are available.

## Conflict of interests

All authors declared no conflict of interests.

## References

[1] Thompson J., Lakhani N. (2015). Cataracts. Prim Care.

[2] Liu X., Guan Z., Liang S., Feng S., Zhou Y. (2024). Associations of cataract, cataract surgery with dementia risk: a systematic review and meta-analysis of 448, 140 participants. Eur J Clin Invest.

[3] Lee C.M., Afshari N.A. (2017). The global state of cataract blindness. Curr Opin Ophthalmol.

[4] Delbarre M., Froussart-Maille F. (2020). Signs, symptoms, and clinical forms of cataract in adults. J Fr Ophtalmol.

[5] Shiels A., Hejtmancik J.F. (2017). Mutations and mechanisms in congenital and age-related cataracts. Exp Eye Res.

[6] Huang P., Hu Y., Duan Y. (2022). TGF-β2-induced circ-PRDM5 regulates migration, invasion, and EMT through the miR-92b-3p/COL1A2 pathway in human lens epithelial cells. J Mol Histol.

[7] Lovicu F.J., Steven P., Saika S., McAvoy J.W. (2004). Aberrant lens fiber differentiation in anterior subcapsular cataract formation: a process dependent on reduced levels of Pax6. Invest Ophthalmol Vis Sci.

[8] Wormstone I.M., Eldred J.A. (2016). Experimental models for posterior capsule opacification research. Exp Eye Res.

[9] Li H., Ji L., Shen H. (2023). The long noncoding RNA H19 promotes fibrotic processes in lens epithelial cells. Invest Ophthalmol Vis Sci.

[10] Neuhann I., Neuhann L., Neuhann T. (2022). Die senile Katarakt [Age-related cataract]. Klin Monbl Augenheilkd.

[11] Gimbel H.V., LeClair B.M., Jabo B., Marzouk H. (2018). Incidence of implantable Collamer lens-induced cataract. Can J Ophthalmol J Can D'ophtalmol.

[12] Li Q., Wang Y., Shi L. (2023). Arginase-1 promotes lens epithelial-to-mesenchymal transition in different models of anterior subcapsular cataract. Cell Commun Signal.

[13] Hales A.M., Chamberlain C.G., McAvoy J.W. (1995). Cataract induction in lenses cultured with transforming growth factor-beta. Invest Ophthalmol Vis Sci.

[14] Gao X.L., Zheng M., Wang H.F. (2019). NR2F1 contributes to cancer cell dormancy, invasion and metastasis of salivary adenoid cystic carcinoma by activating CXCL12/CXCR4 pathway. BMC Cancer.

[15] Tomassy G.S., De Leonibus E., Jabaudon D. (2010). Area-specific temporal control of corticospinal motor neuron differentiation by COUP-TFI. Proc Natl Acad Sci U S A.

[16] Hino-Fukuyo N., Kikuchi A., Yokoyama H. (2017). Long-term outcome of a 26-year-old woman with West syndrome and an nuclear receptor subfamily 2 group F member 1 gene (NR2F_1_) mutation. Seizure.

[17] Li D., Xu M., Wang Z. (2022). The EMT-induced lncRNA NR2F1-AS1 positively modulates NR2F1 expression and drives gastric cancer via miR-29a-3p/VAMP7 axis. Cell Death Dis.

[18] Kim E.J., Kim J.S., Lee S. (2022). ZEB1-regulated lnc-Nr2f1 promotes the migration and invasion of lung adenocarcinoma cells. Cancer Lett.

[19] Rodriguez-Tirado C., Kale N., Carlini M.J. (2022). NR2F1 is a barrier to dissemination of early-stage breast cancer cells. Cancer Res.

[20] Qiao Y., Sun Z., Tan C., Lai J., Sun X., Chen J. (2022). Intracameral injection of AAV-DJ.COMP-ANG1 reduces the IOP of mice by reshaping the trabecular outflow pathway. Invest Ophthalmol Vis Sci.

[21] Liu X., Meng J., Liao X. (2023). A *de novo* missense mutation in MPP2 confers an increased risk of Vogt-Koyanagi-Harada disease as shown by trio-based whole-exome sequencing. Cell Mol Immunol.

[22] Xiao W., Chen X., Li W. (2015). Quantitative analysis of injury-induced anterior subcapsular cataract in the mouse: a model of lens epithelial cells proliferation and epithelial-mesenchymal transition. Sci Rep.

[23] Nathu Z., Dwivedi D.J., Reddan J.R., Sheardown H., Margetts P.J., West-Mays J.A. (2009). Temporal changes in MMP mRNA expression in the lens epithelium during anterior subcapsular cataract formation. Exp Eye Res.

[24] Hou M., Luo F., Ding Y. (2024). Let-7c-3p suppresses lens epithelial-mesenchymal transition by inhibiting cadherin-11 expression in fibrotic cataract. Mol Cell Biochem.

[25] Chen X., Xiao W., Chen W. (2017). MicroRNA-26a and-26b inhibit lens fibrosis and cataract by negatively regulating Jagged-1/Notch signaling pathway. Cell Death Differ.

[26] En A., Takauji Y., Miki K., Ayusawa D., Fujii M. (2020). Lamin B receptor plays a key role in cellular senescence induced by inhibition of the proteasome. FEBS Open Bio.

[27] Bergamini E., Del Roso A., Gori Z., Masiello P., Masini M., Pollera M. (1994). Endocrine and amino acid regulation of liver macroautophagy and proteolytic function. Am J Physiol.

[28] Huang J., Yu W., He Q. (2022). Autophagy facilitates age-related cell apoptosis - a new insight from senile cataract. Cell Death Dis.

[29] Han J., Wang L., Lv H. (2021). EphA2 inhibits SRA01/04 cell apoptosis by suppressing autophagy via activating PI3K/Akt/mTOR pathway. Arch Biochem Biophys.

[30] Sun Y., Xiong L., Wang X. (2021). Autophagy inhibition attenuates TGF-β2-induced epithelial-mesenchymal transition in lens epithelial cells. Life Sci.

[31] Xiang J., Kang L., Gao H. (2019). BLM can regulate cataract progression by influencing cell vitality and apoptosis. Exp Eye Res.

[32] Shiels A., Hejtmancik J.F. (2019). Biology of inherited cataracts and opportunities for treatment. Annu Rev Vis Sci.

[33] Li H., Song H., Yuan X., Li J., Tang H. (2019). miR-30a reverses TGF-β2-induced migration and EMT in posterior capsular opacification by targeting Smad2. Mol Biol Rep.

[34] Li H., Yuan X., Li J., Tang X. (2015). Implication of Smad2 and Smad3 in transforming growth factor-β-induced posterior capsular opacification of human lens epithelial cells. Curr Eye Res.

[35] Liu H., Jiang B. (2020). Let-7a-5p represses proliferation, migration, invasion and epithelial-mesenchymal transition by targeting Smad2 in TGF-b2-induced human lens epithelial cells. J Bio Sci.

[36] Ma B., Yang L., Jing R. (2018). Effects of Interleukin-6 on posterior capsular opacification. Exp Eye Res.

[37] Meng F., Khoso M.H., Kang K. (2021). FGF21 ameliorates hepatic fibrosis by multiple mechanisms. Mol Biol Rep.

[38] Meng M., Tan J., Chen W. (2019). The fibrosis and immunological features of hypochlorous acid induced mouse model of systemic sclerosis. Front Immunol.

[39] Montero P., Milara J., Roger I., Cortijo J. (2021). Role of JAK/STAT in interstitial lung diseases; molecular and cellular mechanisms. Int J Mol Sci.

[40] Wang J., Ge S., Wang Y. (2021). Puerarin alleviates UUO-induced inflammation and fibrosis by regulating the NF-κB P65/STAT3 and TGFβ1/smads signaling pathways. Drug Des Dev Ther.

[41] You X., Jiang X., Zhang C. (2022). Dihydroartemisinin attenuates pulmonary inflammation and fibrosis in rats by suppressing JAK2/STAT3 signaling. Aging.

[42] Zhang Y., Zhang L., Fan X. (2019). Captopril attenuates TAC-induced heart failure via inhibiting Wnt3a/β-catenin and Jak2/Stat3 pathways. Biomed Pharmacother.

[43] Dees C., Pötter S., Zhang Y. (2020). TGF-β-induced epigenetic deregulation of SOCS_3_ facilitates STAT3 signaling to promote fibrosis. J Clin Invest.

[44] Kasembeli M.M., Bharadwaj U., Robinson P., Tweardy D.J. (2018). Contribution of STAT3 to inflammatory and fibrotic diseases and prospects for its targeting for treatment. Int J Mol Sci.

[45] Marconi G.D., Fonticoli L., Rajan T.S. (2021). Epithelial-mesenchymal transition (EMT): the type-2 EMT in wound healing, tissue regeneration and organ fibrosis. Cells.

[46] Nieto M.A., Huang R.Y.J., Jackson R.A., Thiery J.P. (2016). EMT: 2016. Cell.

[47] Liu H., Takagaki Y., Kumagai A., Kanasaki K., Koya D. (2021). The PKM2 activator TEPP-46 suppresses kidney fibrosis via inhibition of the EMT program and aberrant glycolysis associated with suppression of HIF-1α accumulation. J Diabetes Investig.

[48] Bertacchi M., Tocco C., Schaaf C.P., Studer M. (2022). Pathophysiological heterogeneity of the BBSOA neurodevelopmental syndrome. Cells.

[49] Bonzano S., Dallorto E., Molineris I. (2023). NR2F1 shapes mitochondria in the mouse brain, providing new insights into Bosch-Boonstra-Schaaf optic atrophy syndrome. Dis Model Mech.

[50] Liang Q., Xu Z., Liu Y. (2022). NR2F1 regulates TGF-β1-mediated epithelial-mesenchymal transition affecting platinum sensitivity and immune response in ovarian cancer. Cancers.

[51] Zou S., Tong Q., Liu B., Huang W., Tian Y., Fu X. (2020). Targeting STAT3 in cancer immunotherapy. Mol Cancer.

[52] Sadrkhanloo M., Entezari M., Orouei S. (2022). STAT3-EMT axis in tumors: modulation of cancer metastasis, stemness and therapy response. Pharmacol Res.

[53] Jin W. (2020). Role of JAK/STAT3 signaling in the regulation of metastasis, the transition of cancer stem cells, and chemoresistance of cancer by epithelial-mesenchymal transition. Cells.

[54] Wang L.N., Zhang Z.T., Wang L. (2022). TGF-β1/SH2B3 axis regulates anoikis resistance and EMT of lung cancer cells by modulating JAK2/STAT3 and SHP2/Grb2 signaling pathways. Cell Death Dis.

[55] Zhang X., Sai B., Wang F. (2019). Hypoxic BMSC-derived exosomal miRNAs promote metastasis of lung cancer cells via STAT3-induced EMT. Mol Cancer.

[56] Wei C., Yang C., Wang S. (2019). Crosstalk between cancer cells and tumor associated macrophages is required for mesenchymal circulating tumor cell-mediated colorectal cancer metastasis. Mol Cancer.

[57] Shen M., Xu Z., Xu W. (2019). Inhibition of ATM reverses EMT and decreases metastatic potential of cisplatin-resistant lung cancer cells through JAK/STAT3/PD-L1 pathway. J Exp Clin Cancer Res.

[58] Shu D.Y., Lovicu F.J. (2017). Myofibroblast transdifferentiation: the dark force in ocular wound healing and fibrosis. Prog Retin Eye Res.

