# Supplementary material for: Autophagy-induced NR2F1 activation promotes the apoptosis of lens epithelial cells and facilitates cataract-associated fibrosis through targeting STAT3
Source: Genes Dis. 2025 Jan 28;12(5):101549. doi: 10.1016/j.gendis.2025.101549 (PMC12242405; doi:10.1016/j.gendis.2025.101549)
Supplement: Multimedia component 1 [file mmc1.docx]

**Supplementary fig. 1.** The immunofluorescence intensity of NR2F1 in TGF-β1-induced SRA01/04 cells with different concentration of chloroquine. (Scar bar: 50 μm).

**Supplementary fig. 2.** The knockdown efficiency of NR2F1 in mice lens treated with AAV-NR2F1.

**Supplementary table 1.** The Antibodies for western blotting used in this study.

**sFig. 1**


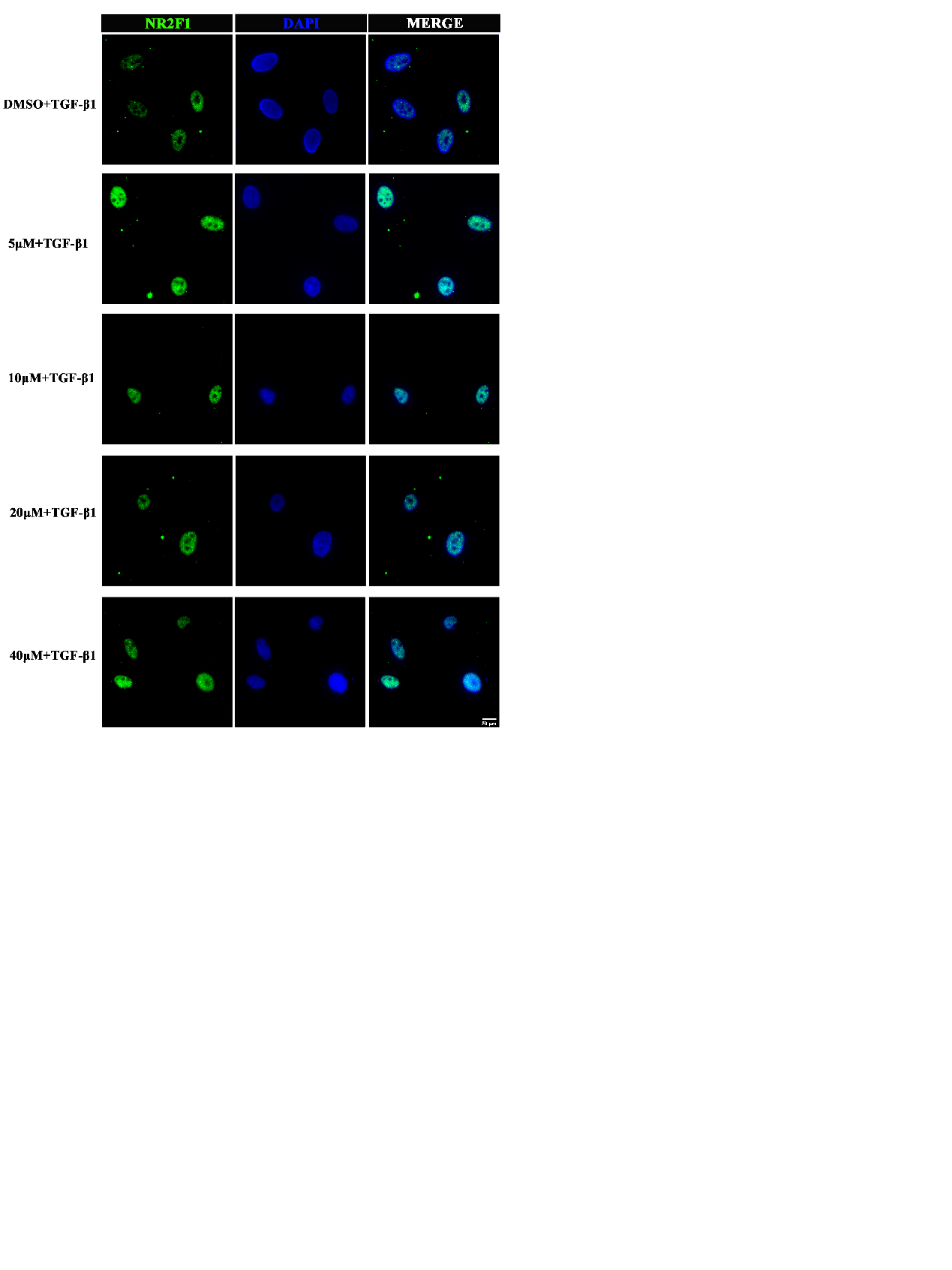


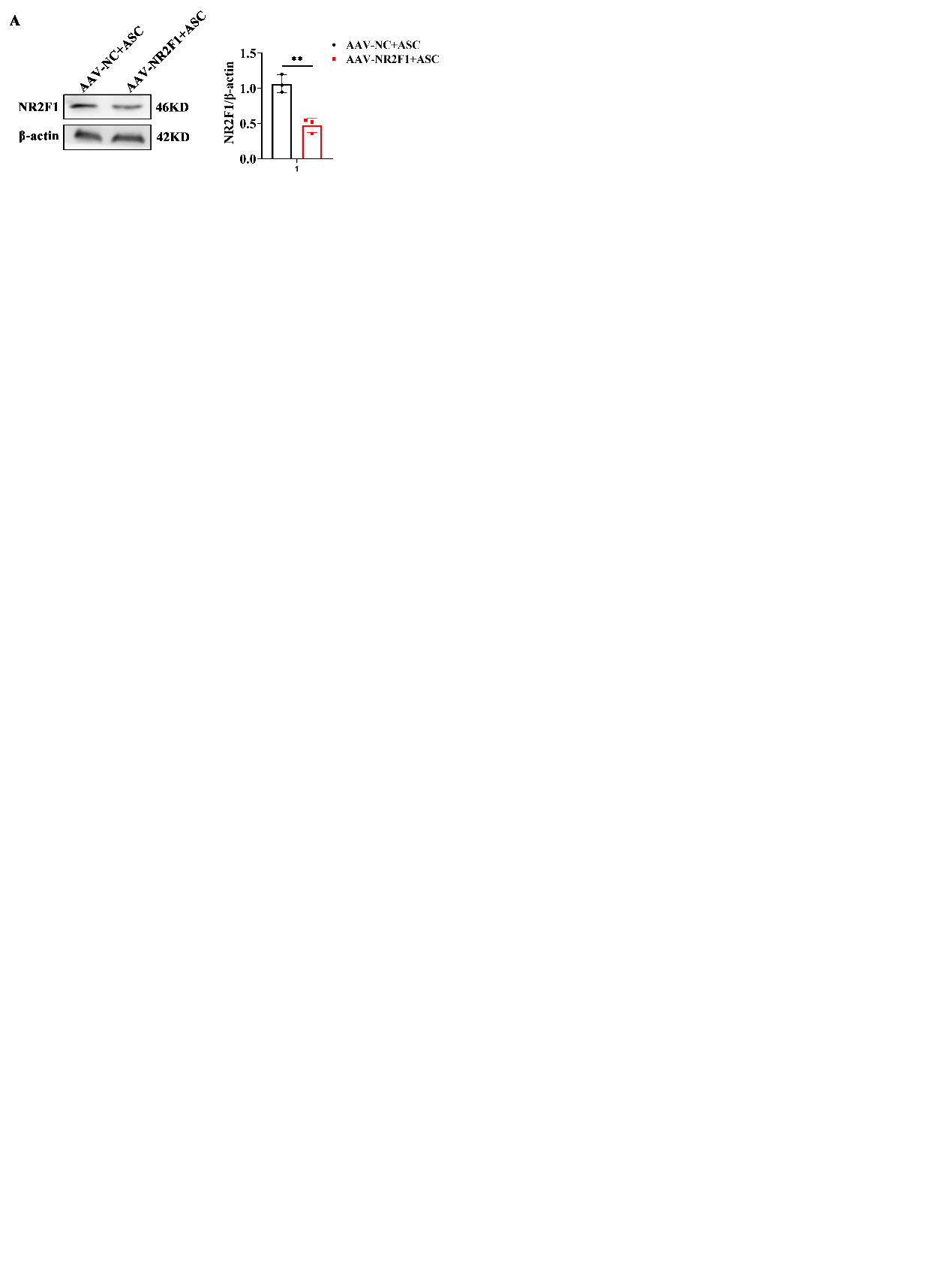
**sFig. 2**

**Supplementary table 1 The Antibodies for western blotting used in this study**

| **Antibody** | **Host** | **Manufacturer** | **Application** |
| --- | --- | --- | --- |
| NR2F1 | Rabbit | Abcam | 1:1000 |
| FN1 | Rabbit | Proteintech | 1:1000 |
| VIMENTIN | Rabbit | Huabio | 1:1000 |
| α-SMA | Rabbit | Abcam | 1:1000 |
| CASPASE3 | Mouse | Proteintech | 1:1000 |
| BAX | Mouse | Proteintech | 1:1000 |
| JAK1 | Rabbit | Huabio | 1:500 |
| STAT3 | Rabbit | Abcam | 1:1000 |
| P-STAT3 | Rabbit | abcam | 1:1000 |
| SMAD2  MYD88  β-actin | Rabbit  Rabbit  Rabbit | Wanlei  Proteintech  Affinity | 1:1000  1:800  1:5000 |
